# Validation of the Sour Seven Questionnaire for screening delirium in hospitalized seniors by informal caregivers and untrained nurses

**DOI:** 10.1186/s12877-016-0217-2

**Published:** 2016-02-15

**Authors:** Richard W. Shulman, Saurabh Kalra, Joanne Zhuan Jiang

**Affiliations:** Trillium Health Partners, Queensway Health Centre, 150 Sherway Drive, 4th floor, Toronto, ON M9C 1A5 Canada; Faculty of Medicine, Department of Psychiatry, Division of Geriatric Psychiatry, University of Toronto, Toronto, Canada; Mississauga Academy of Medicine, University of Toronto, Mississauga, Canada; Internal Medicine, University of Toronto, Toronto, Canada

**Keywords:** Delirium, Hospitalized seniors, Screening tools, Delirium detection

## Abstract

**Background:**

Delirium is a common condition in hospitalized seniors that nonetheless often goes undetected by nurses or is delayed in being detected which negatively impacts quality of care and outcomes. We sought to develop a new screening tool for delirium, The Sour Seven Questionnaire, a 7-item questionnaire suitable to be completed from informal or untrained caregiver observation. The study aimed to develop the scoring criteria for a positive delirium screen and assess concurrent validity of the questionnaire against a geriatric psychiatrist’s assessment.

**Methods:**

A pilot study of 80 hospitalized seniors over age 65 recruited from three units (2 medical, 1 orthopedic). Participants were assessed using the Confusion Assessment Method (CAM) with a brief cognitive screen and the Sour Seven Questionnaire posed to the appointed informal caregiver (family member) or untrained nurse for up-to 7 days. Subjects testing positive on the CAM and a random sample of negatively CAM screened subjects were assessed by the geriatric psychiatrist.

**Results:**

From 80 participants, 21 screened positive for delirium on the CAM. 18 of the 21 CAM positive screens were diagnosed to have delirium by the geriatric psychiatrist, and 17 of the 18 randomly assigned negative CAM screens were confirmed as not having delirium. From the questionnaires on these 39 participants, weighted scoring for each of the 7 questions of the Sour Seven Questionnaire was developed based on their relative risks for correctly predicting delirium when compared to the geriatric psychiatrist’s clinical assessment. Total scoring of the questionnaire resulted in the following positive predictive values for delirium: 89 % with a total score of 4 (sensitivity 89.5 %, specificity 90 %), and 100 % with a total score of 9 (sensitivity 63.2 %, specificity 100 %). Comparison between scoring on questionnaires posed to informal caregivers versus untrained nurses showed no differences.

**Conclusion:**

A weighted score of 4 in the Sour Seven Questionnaire has concurrent validity as a screening tool for delirium and a score of 9 is diagnostic for delirium. The Sour Seven Questionnaire is the first screening tool for delirium shown to be suitable for use by informal caregivers and untrained nurses in hospitalized seniors.

**Electronic supplementary material:**

The online version of this article (doi:10.1186/s12877-016-0217-2) contains supplementary material, which is available to authorized users.

## Background

Delirium in hospitalized seniors is a state of confusion with an acute onset that develops over a few hours to days characterized by inattentiveness and disturbances in consciousness, orientation, memory, thought, perception, and behaviour [1]. Prevalence of delirium in seniors on admission to a hospital is estimated at 14 to 24 %, and incidence of delirium arising during hospitalization ranges from 6 to 56 % [2]. Delirium is associated with substantial morbidity, increased average length of stay, functional decline, persistent cognitive impairment, loss of independence, higher Long Term Care (LTC) institutionalization rates, worse rehabilitation outcomes and death with an in-hospital mortality of up to 33 % [3, 4].

Effective detection of delirium in hospitalized seniors remains problematic. Overall non-detection rates of delirium in hospitalized seniors have been reported to range from 33 % to as high as 66 % [5]. It is challenging for health care professionals to detect delirium in people with depression and particularly dementia because of overlapping symptoms [6].

Similarly, we believed there was a considerable lack of appropriate detection of delirium at the Mississauga Hospital, part of Trillium Health Partners (THP), a large academically affiliated community hospital in Mississauga, Ontario. According to the hospital’s Health Records Department, from April to December in 2010, of 8569 admitted seniors (age 65 or over) only 290 (3.4 %) were diagnosed with delirium on admission and only 492 (5.7 %, mean age 82) were given a diagnosis of delirium during their hospital stay. Despite a pilot quality improvement education project conducted during the summer of 2011 to improve nurses’ detection of delirium using the Confusion Assessment Method (CAM) [7] (arguably the gold standard bedside delirium screening tool [8]) used with a cognitive assessment tool (the Sweet 16 [9] since removed from use following alleged copyright infringement [10]) to ensure maximum CAM effectiveness, we nonetheless found low nursing compliance and no improvement in rates of delirium detection [11]. We found that nurses untrained in mental health were uncomfortable assessing key features of the CAM despite providing them with a delirium educational program. Frequent complaints particularly included lack of familiarity with the patient needed to assess acute onset of confusion and/or fluctuating course, and inability or unwillingness to conduct the cognitive assessment required for an effective assessment of inattentiveness required by the CAM. Limitation of detection of delirium by nurses using the CAM has also been previously demonstrated [12].

To our knowledge there have not been any validated tools for delirium screening by informal family caregivers in hospitalized seniors. A version of the CAM, the Family-CAM (FAM-CAM) [13] was developed based on the CAM, to provide a method for informant-based assessment of delirium by interviewing caregivers either in person, on the telephone, or electronically. Features assessed include: acute changes in concentration, inattention, disorganized speech, excessive drowsiness, disorientation, perceptual disturbances and inappropriate behaviour. The FAM-CAM is a sensitive screening tool (sensitivity of 88 % and specificity of 98 % against the CAM) but validated so far only in a community setting [14].

We hypothesized that both untrained nurses and informal caregivers (family members) could be of assistance in screening for delirium in hospitalized seniors if provided a simple means for identifying signs of delirium. Therefore we sought to create a novel questionnaire for untrained nurses and/or informal caregivers that would assist in the detection of delirium in hospitalized seniors that requires no training, no prior knowledge of the patient, no questions posed to the patient, is independent of language, and could be suitable for those patients with dementia (chronic confusion) based on seven simple observations of the patient during caregiving.

## Methods

### Creation of the tool

We began by examining available screening tools for delirium detection [15–17]. At least 24 such scales have been identified that vary in their purpose, method of data collection, the rater, number of items, scoring and the time required for rating. The tools we looked at particularly included the CAM, the NEECHAM Confusion Scale [18], the Nursing Delirium Screening Scale (Nu-DESC) [19], the Delirium Observation Scale (DOS) [20, 21], the Delirium Symptom Interview [22], the Memorial Delirium Assessment Scale [23], the Clinical Assessment of Confusion-A (CAC-A) [24], and B (CAC-B) [25], the Saskatoon Delirium Checklist [26], the Delirium Rating Scale-Revised-98 (DRS-R-98) [27], and the Delirium Index (used to rate severity of delirium) [28]. These tools while comprehensive in their assessment criteria for delirium were nonetheless found to be inadequate for our purposes. Common problems included having to assess the patient’s mental state in some way, too many questions or taking too long to complete, use of medical terminology or phrases not normally understood by untrained caregivers, and overlap of signs observed in patients with dementia without delirium. To ensure practicality of use in dementia patients, we focused on those features of delirium that do not overlap with dementia and are potentially easier to recognize by untrained caregivers.

We decided upon seven behavioural questions of which four reflect the DSM-IV [29] and subsequent DSM-5 [30] diagnostic criteria of delirium, including disturbances in level of *awareness*, level of *attentiveness*, *fluctuations* in awareness and attentiveness, and *disordered thinking*. Similarly these are the essential components of the CAM and we believe would be required elements of any delirium detection tool. Additionally we added three criteria not found in the CAM or DSM criteria. These included disorganized behaviour similarly listed in several other screening tools, but also two novel criteria not specifically found in other screening tools. The last two criteria enquire about functional disruption in activities of daily living; (ADLs) [31] – conceptualized by the actions required in walking, toileting, grooming, bathing, selecting proper attire, dressing, and feeding. A strong relationship between the presence of delirium and worsening physical function in ADLs in seniors in hospital and in 3 month follow–up has been previously demonstrated [32].

We chose unexplained difficulty with feeding oneself and unexplained difficulty with mobility (or movement if the person is immobile) rather than toileting, grooming, bathing or dressing, as disruption in these latter ADLs could be accounted for by various other factors in hospitalized seniors. Unexplained difficulty with feeding oneself can be differentiated from apraxia of feeding oneself in advanced dementia as it must be unexplained. Unexplained difficulty with mobility relates to data that falls in general hospital inpatients are associated with delirium and advanced age [33]. This question is distinct from the typically asked questions about psychomotor agitation or retardation which may not be easily distinguishable from motor symptoms seen in dementia patients. For the same reason of needing to distinguish signs of delirium from signs of dementia, there are no questions about disorientation, hallucinations, delusions, inappropriate behaviour, safety, insomnia, mood, or memory. We specifically wished to avoid the need for the untrained caregiver to have had any prior knowledge of the patient such as commonly encountered by nurses. For this reason, there are no questions enquiring about acute onset of symptoms. Similarly to avoid the need to assess the patient’s mental status, there are no questions to formally assess cognition, attention or speech. The Sour Seven Questionnaire can be downloaded from Additional file 1. 

### Proof of concept demonstration study

An initial proof of concept demonstration study was conducted to verify that our questionnaire had the potential feasibility for real-world application, but not to determine validity of the questionnaire which would then be addressed in the pilot study. The student researcher (J.J.) interviewed nurses caring for patients aged 65 and over on three hospital units (1 surgical and 2 medical) with the Sour Seven Questionnaire daily for 5 weeks during the summer of 2012. Each individual interview took 1 to 2 min. The responses to the 7 questions on the Sour Seven Questionnaire were based on the nurses’ interactions with the patient since the beginning of their shift. Nurses’ responses were kept confidential. Research ethics approval was obtained prior to initiation of the study from the THP Research Ethics Board, and individual nurse’s consent was obtained. The goal of the study was to evaluate the feasibility of the questionnaire based on the student’s interactions with the nurses.

All nurses approached except for one consented to being interviewed, and none withdrew from the study suggesting that there was minimal objection to the small additional task to their daily care routine. A total of 271 questionnaires were completed relating to 86 elderly inpatients during the 5 week study. Compliance with completing the questionnaire interview was excellent. We found that the Sour Seven Questionnaire was non-threatening for untrained caregivers. The individual questions are written to be as straight forward and brief as possible to ensure the questionnaire will fit on 1 page of standard letter sized paper.

The research student’s qualitative observations were that questions regarding observation of patient alertness, attentiveness, fluctuations and disorganized behaviour were easiest to answer whereas nurses experienced challenges in interpreting disordered thinking particularly when there was a language barrier present. Questions pertaining to nurses’ observation of impaired eating/drinking and mobility/movement were most challenging to answer. We suggest this was due to nurses being less involved in these ADLs during routine care in these patients. Functional decline in these ADLs during hospitalization may be better observed by allied healthcare workers providing caregiving such as occupational, physical and/or speech-language therapists; or informal caregivers including personal support workers or family members. As a result, we planned the pilot validation study for the Sour Seven Questionnaire in hospitalized seniors to be done with both nurses and informal (family member) caregivers. The pilot project study was conducted during the summer of 2013 with the objective to develop the scoring criteria for a positive delirium screen and to assess the concurrent validity of the Sour Seven Questionnaire against a geriatric psychiatrist’s (R.W.S.) assessment.

### Study sample for validation

The eligible participants for this study were English-speaking seniors above the age of 65 who were admitted to either the medical or surgical units at the Mississauga Hospital and were staying at the hospital for at least one day. Informed written consent was provided by all participants or their substitute decision makers if the participants were incapable of doing so. The study received approval from the THP Research Ethics Board (ID # 571).

### Study design

The study was conducted between June to August 2013. Each morning, the medical student (S.K.) visited the potential participants meeting the age criteria. While explaining the study, all participants were given an option to assign a caregiver who would be interviewed to answer the items on the Sour Seven Questionnaire. The caregiver was any individual who was visiting the patient at the hospital during their admission for at least 2 h in a day. If the participant did not wish to assign a caregiver, they were given the option to have their nurse for the day answer the questions on the Sour Seven Questionnaire.

The CAM assessments were done on a daily basis during the participants’ stay at the hospital by the student investigator (S.K.) who assessed the participant with the CAM combined with a brief cognitive screen (orientation, registration, digit span testing and short term recall). The student investigator received one-on-one training in administering CAM and the cognitive screen by the geriatric psychiatrist. The diagnostic algorithm for CAM has 4 cardinal features of delirium: 1) acute onset and fluctuating course, 2) inattention, 3) disorganized thinking, and 4) altered level of consciousness. A positive CAM screen in this study is defined as meeting the criteria (1) or (2) and either one of (3) or (4). This maximizes the CAM’s ability to screen for delirium resulting in a higher sensitivity and lower specificity with minimizing the number of false negatives [34].

The student investigator (S.K.) also posed the Sour Seven Questionnaire to the appointed caregiver during a 2 to 5 min interview in which the 7 questions were read verbatim to them and their responses were recorded. The CAM and Sour Seven Questionnaires were done for a maximum of 7 days or until the participant screened positive for delirium on the CAM. All subjects that screened positive for delirium on the CAM and a random sample of CAM negative screens were assessed by the geriatric psychiatrist (R.W.S.) on the same or next day using the DSM-5 criteria for a diagnosis of delirium. The geriatric psychiatrist was blinded to the student investigator’s assessment in order to prevent any bias. If the geriatric psychiatrist was unable to assess the participants on the same day as the CAM and Sour Seven screening, then the clinical chart was also reviewed to detect any documentation of delirium symptoms by nurses or physicians the day before. For this study, the geriatric psychiatrist’s assessment was considered to be the gold standard against which the Sour Seven Questionnaire was validated.

### Statistical analysis

The statistical analysis was performed using SAS Statistical Analysis Software. The analysis was performed using the data of the participants that completed the two screens and the geriatric psychiatrist’s assessment. For each of the 7 questions, logistic regression was done to determine the probability of the geriatric psychiatrist’s assessment being “yes” for delirium when the answer to a given Sour Seven question is “yes” or “no”. The logistic regression results were expressed as relative risks (RR) along with 95 % CI and c-statistics. The validation characteristics (sensitivity, specificity, positive and negative predictive values and Youden index) were determined for each score and a receiver operating characteristic (ROC) curve was constructed to determine cutoffs for a positive delirium screen. Finally, a comparison between the assessments of the nurses and informal caregivers was done for each question using the Fisher’s exact test. For all calculations, a 2-tailed *p*-value of <0.05 was considered to be statistically significant.

## Results

A total of 80 participants were recruited into this study and all of them went through CAM screening and Sour Seven Questionnaires. The prevalence of delirium in this study was 22.5 %. The final analysis for determining the validation characteristics of the Sour Seven Questionnaire was done on 39 participants who completed all 3 assessments. Table 1 shows the baseline demographic characteristics of the study participants, both in the analyzed and non-analyzed groups. The mean age of participants was 81.3 ± 8.9 years in the analyzed group and 79.2 ± 10.2 in the non-analyzed group. The majority of the participants were females (64 % in analyzed and 61 % in non-analyzed group).

**Table 1:** Baseline demographic characteristics of participants

Characteristic	Analyzed group (*n* = 39)	Non-analyzed group (*n* = 41)
Age in years (mean ± SD)	81.3 ± 8.9	79.2 ± 10.2 years
Sex n (%)		
Male	14 (36 %)	15 (37 %)
Female	25 (64 %)	26 (63 %)
Patient location n (%)		
Medicine	18 (46 %)	16 (39 %)
Surgery	21 (54 %)	25 (61 %)

Twenty one participants screened positive for delirium on the CAM, and 18 of these were found to have delirium by the geriatric psychiatrist. Of the negative CAM screens, 18 out of 69 (26 %) were randomly assigned for geriatric psychiatrist’s assessment. Seventeen of 18 negative CAM screen cases randomly assigned for assessment were confirmed as not having delirium by the geriatric psychiatrist. The sensitivity of the CAM was 94.7 % and specificity was 85 %.

Table 2 shows the results from the logistic regression analysis for each of the 7 questions in the Sour Seven Questionnaire, including the relative risks and 95 % Confidence Intervals, and the corresponding c-statistics. The RR for each question were rounded to the nearest whole number and used to determine a weighted scoring criteria. The 95 % CI showed statistical significance (*p* < 0.05) for all questions except for Question #7.

**Table 2:** Relative risks, c-statistics and weighted scores of Sour Seven Questions to predict delirium in comparison with geriatric psychiatrist’s assessment

Question #	Relative risk (95 % CI)	c-statistic	Weighted score
1 (Altered level of awareness)	3.43 (1.79–6.58)	0.79	3
2 (Reduced attentiveness)	3.87 (1.90–7.89)	0.62	4
3 (Fluctuation)	3.43 (1.79–6.58)	0.79	3
4 (Disordered thinking)	2.61 (1.52–4.49)	0.71	3
5 (Disorganized behaviour)	2.42 (1.44–4.09)	0.69	2
6 (Impaired eating/drinking)	1.82 (1.82–1.82)	0.63	2
7 (Difficulty in mobility)	1.27 (0.57–2.84)	0.53	1
Total			18

**Fig. 1:**
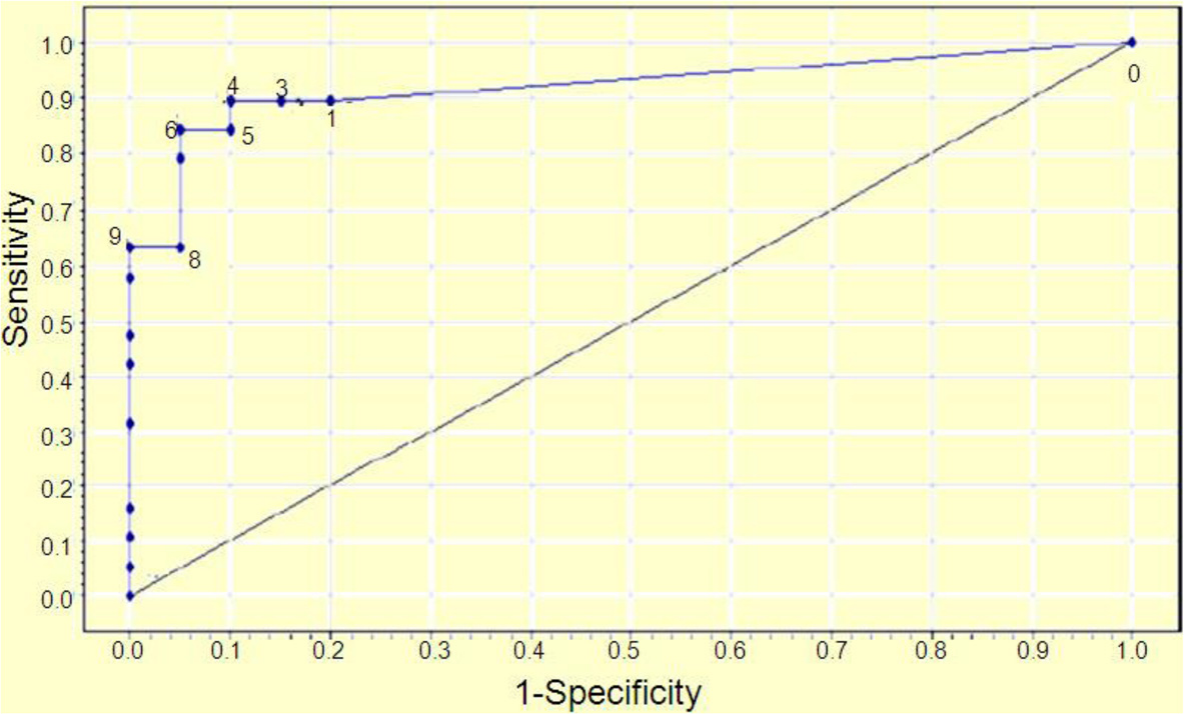
Receiver Operating Characteristic (ROC) Curve using weighted scores in the Sour Seven Questionnaire (Area under the curve = 0.921)

The maximum total score in the Sour Seven Questionnaire is 18. Figure 1 shows the Receiver Operating Characteristic (ROC) curve with data points for each of the scores from 1 to 18. The validation characteristics (sensitivity and specificity) for each score were analyzed to determine scores for a positive delirium screen. Scores of 4 and 9 were used to classify patients as having possible delirium, and delirium respectively. The score of 4 was selected as the screening cut-off because it has a higher Youden Index than a score of 3 or 5, and the score of 9 was selected as diagnostic of delirium because of its specificity of 100 % and high Youden Index. The validation characteristics of these scores are summarized in Table 3.

**Table 3:** Validation characteristics used to determine classification scores

Total score	Sensitivity	Specificity	PPV	NPV	Youden index	Classification
3	89.5 %	85.0 %	85.0 %	89.4 %	0.745	
4	89.5 %	90.0 %	89.5 %	90.0 %	0.795	possible delirium
5	84.2 %	90.0 %	88.9 %	85.7 %	0.742	
6	84.2 %	95.0 %	94.1 %	86.4 %	0.792	
7	78.9 %	95.0 %	94.0 %	82.0 %	0.739	
8	63.2 %	95.0 %	92.3 %	73.1 %	0.582	
9	63.2 %	100.0 %	100.0 %	74.1 %	0.632	delirium
10	57.9 %	100.0 %	100.0 %	71.0 %	0.579	

*PPV* Positive Predictive Value, *NPV* Negative Predictive Value

For each of the 7 questions, the Fisher exact test analysis was done to compare the proportion of agreement between the questionnaires posed to untrained nurses against the geriatric psychiatrist assessment versus the questionnaires posed to informal caregivers against the geriatric psychiatrist (Table 4). The *p* value from this analysis for each of the 7 questions was greater than 0.05, suggesting that there was no difference between the questionnaires posed to nurses versus informal caregivers.

**Table 4:** Comparison of agreement between nurses and geriatric psychiatrist versus caregivers and geriatric psychiatrist

Question	% Agreement between nurses and MD Dx. (%)	% Agreement between caregivers and MD Dx. (%)	*P* value of fisher exact test
1	12/14 (85.7 %)	19/25 (76 %)	0.69
2	11/14 (78.5 %)	21/25 (84 %)	0.69
3	12/14 (85.7 %)	19/25 (76 %)	0.69
4	13/14 (92.8 %)	15/25 (60 %)	0.06
5	11/14 (78.5 %)	16/25 (64 %)	0.48
6	11/14 (78.5 %)	13/25 (52 %)	0.17
7	9/14 (64.3 %)	11/25 (44 %)	0.32

MD Dx. Geriatric Psychiatrist’s diagnosis of delirium

*P* value greater than 0.05 suggests no difference between the questionnaires posed to nurses versus informal caregivers

## Discussion

Results of this pilot study suggest a weighted ability of the questions to validly predict delirium, with Questions # 1 to 4 having the highest weighted score (each being 3 or 4), while Questions # 5 to 7 having low to moderate weighted scores (1 or 2). This is not surprising since the delirium characteristics assessed by Questions 1 to 4, such as altered level of awareness, inattentiveness, fluctuating course and disordered thinking are also present in the DSM-5 criteria and various other delirium screening and diagnostic instruments. In contrast, the characteristics assessed by Questions 5 to 7 aimed at capturing the functional impact of delirium were found to have a lower ability to predict delirium. This may be partially due to the small sample size resulting in a low study power. Although Question 7’s predictive ability for delirium based on unexplained difficulty with mobility or movement was not statistically significant, it was still kept in the questionnaire and given a lower weighting as the lack of statistical significance may have been because the participants on the hospital units may have had restricted mobility due to their acute illness and/or post-operative states. It may be that in alternative settings such as Long Term Care (LTC) Homes or the Emergency Department (ED), this question may have more applicability.

The cut-off scores of 4 and 9 were developed from the ROC curve and the Youden index to classify patients as having possible delirium, and delirium respectively. As expected, higher cutoff scores resulted in increasing specificity and decreasing sensitivity because a higher cutoff decreases the number of false positives. In order to use the Sour Seven Questionnaire as a screening instrument, a high sensitivity is desired which is achieved with a score of 4. On the other hand, a score of 9 has high specificity. This indicates that a score greater than or equal to 9 is strongly suggestive of the diagnosis of delirium. Scores below 9 but above a cut off of 4 on the Sour Seven Questionnaire suggest the need for comprehensive assessment to rule out delirium.

We confirmed that the Sour Seven Questionnaire can be completed both by informal caregivers without any prior medical training as well as untrained nurses without prior knowledge of the patient. This lends support to the utility of the questionnaire in this population and increases the external validity (generalizability) of the results. The questions can be answered by any non-mental health caregiver regardless of training or prior patient knowledge.

Consequently, we argue that informal caregivers can provide a helpful role to aid in delirium detection in hospitalized seniors if given the right tool. Although there have been strategies proposed for informal caregivers to assist in delirium prevention in hospitalized elderly patients [35, 36], there is no tool validated for use by informal caregivers to help with delirium detection in hospitalized seniors. The only other tool we are aware of specifically developed for informal caregivers, the FAM-CAM [13] has been studied only in an outpatient setting, but nonetheless provides supporting evidence that informal caregivers may assist in delirium detection [14]. One of the strengths of our tool is that caregivers do not require instruction in the use of the Sour Seven in contrast to the FAM-CAM which does require extensive instruction. More recently, a single screening question for delirium detection by informal caregivers was compared against the CAM in a hospitalized elderly population [37]. The use of the screening question “How has your relative/friend’s memory changed with his/her current illness?” was found to have only a sensitivity of 77 % and specificity of 56 %, suggesting that this simple screening question by informal caregivers by itself is insufficient to assist in delirium detection. Rather the Sour Seven Questionnaire for assisting delirium detection in hospitalized seniors by informal caregivers offers a high sensitivity and specificity.

The potential limitations of our study include a relatively small and homogeneous English-speaking study population since all our participants were recruited from one hospital site. However, the hospital serves a diverse range of patients and this limitation may have been partially offset by recruiting both medical and surgical patients. Future studies should include a larger sample size and may be done in multiple sites such as LTC or the ED to support the utility of this tool. Secondly, the recruitment in this study was only based on the participants’ age and it was not determined if they had a pre-existing diagnosis of dementia. Future studies should look at the validity of the tool in those with pre-existing dementia in comparison to those with intact premorbid cognition to study the sensitivity and specificity of the Sour Seven Questionnaire tool specifically in those with pre-existing dementia. We also did not classify patients with delirium into hypoactive, hyperactive and mixed types and cannot make any conclusions on the predictive power of the Sour Seven Questionnaire in each delirium subtype. Also, the delirium assessment using the Sour Seven although completed the same or next day, was not done simultaneously as the geriatric psychiatrist’s assessment which may affect the results given the fluctuating course of delirium. Studies in the future may make use of greater staff and resources to ensure that the different assessments are done as close to each other as possible. Furthermore, the questionnaire items were read to the caregivers by the student investigator which may have biased the results. This can be eliminated in future studies by giving paper copies of the questionnaire to the caregivers that they can complete on their own. We do not anticipate difficulties with compliance with independent use. Finally, we also did not evaluate the inter-rater reliability of the Sour Seven Questionnaire. Future studies could look at the inter-rater reliability amongst nurses and informal caregivers and/or between informal caregivers and nurses.

## Conclusion

The prevalence of delirium in our study population of elderly hospitalized patients was 22.5 %. This is consistent with prior studies and in keeping with our assumption that delirium is under-reported at the Mississauga Hospital (Health Records reported prevalence 5.7 %). Clearly our experience is similar to others in that despite being a medical emergency, the presence of delirium often goes unrecognized by health care providers during routine care in a hospital setting [2]. Reasons for lack of delirium detection include: lack of routine formal screening, high prevalence of pre-morbid dementia in hospitalized seniors confounding mental status assessments, language barriers, lack of prior knowledge of the patient by medical staff, lack of nursing training in mental status assessment and geriatric psychiatry, nursing discomfort with cognitive assessments, fluctuation of symptoms during the day, and different clinical presentations of delirium (hypoactive and hyperactive).

Informal caregivers and untrained nurses can provide a helpful role to assist in delirium detection in hospitalized seniors if given the right tool. The results of this pilot study support the concurrent validity of the Sour Seven Questionnaire with high sensitivity and specificity to assist in delirium detection. We conclude that the Sour Seven Questionnaire; a novel, brief, easy to use clinical tool that is consistent with DSM-5 criteria, is a valid screening instrument for assisting delirium detection in hospitalized seniors by any informal or untrained formal caregiver. The Sour Seven Questionnaire is available for open access distribution to be used freely among researchers, clinicians, allied health staff, and all informal caregivers.

## Additional file

Additional file 1:
**The Sour Seven: Delirium Detection Questionnaire for Caregivers.** (PDF 101 kb)
