# Supplementary material for: Validation of the Sour Seven Questionnaire for screening delirium in hospitalized seniors by informal caregivers and untrained nurses
Source: BMC Geriatr. 2016 Feb 15;16:44. doi: 10.1186/s12877-016-0217-2 (PMC4754883; doi:10.1186/s12877-016-0217-2)
Supplement: Additional file 1: — The Sour Seven: Delirium Detection Questionnaire for Caregivers. (PDF 101 kb) [file 12877_2016_217_MOESM1_ESM.pdf]

<u>The Sour Seven</u>: Delirium Detection Questionnaire for Caregivers

The Sour Seven: A questionnaire designed for caregivers to screen for delirium (acute confusion) in seniors, including those with dementia (chronic confusion), that requires no training, no prior knowledge of the person, no questions posed to the person, is independent of language, based on seven simple observations of the person during caregiving.

During your interaction with the person today, have you observed any of the following? Circle the corresponding value in the answer boxes.	YES	NO
1. Altered level of awareness to the environment in any way different than being normally awake.	**3**	0
2. Reduced attentiveness; inability to focus on you during the interaction.	**4**	0
3. Fluctuation in awareness and attentiveness, such as drifting in and out during an interaction or through the day.	**3**	0
4. Disordered thinking; the response (whether verbal or action) is unrelated to the question or request.	**3**	0
5. Disorganized behaviour; purposeless, irrational, under-responsive or over-responsive to requests.	**2**	0
6. Unexplained impaired eating or drinking (excluding appetite); unable to perform the actions to feed oneself.	**2**	0
7. Unexplained difficulty with mobility or movement.	**1**	0
**Score**		

<u>Score</u>	Predictive Value	Description
**4**	89%	**possible delirium**: evaluate potential medical causes, meds/substances
**9**	100%	**delirium**: immediate medical evaluation required

COPYRIGHT ©2014 DR. RICHARD W. SHULMAN, TRILLIUM HEALTH PARTNERS (THP). ALL RIGHTS RESERVED.

This instrument cannot substitute for medical advice, diagnosis or treatment by a trained medical professional. Diagnosis and treatment should be based collectively on medical history and examination along with a health practitioner’s professional judgement and review of all test results. The material contained in this instrument does not contain standards that are meant to be applied rigidly and followed in virtually all cases. A health practitioner’s judgment must remain central to the selection of diagnostic tests and therapy options of a specific patient’s medical condition. This instrument is provided as is, without representation as to its fitness for any purpose, and without warranty of any kind, either express or implied, including without limitation the implied warranties of merchantability and fitness for a particular purpose. Neither Dr. Richard Shulman nor THP has any obligation to provide support, updates, enhancements or other modifications. No guarantees are made with respect to accuracy, completeness, errors, or omissions of content. In no event will Dr. Richard Shulman or THP be liable for any decision made or action taken in reliance upon the information provided through this instrument. Neither Dr. Richard Shulman nor THP shall be liable for any direct, indirect, special, incidental, consequential, punitive or exemplary damages, with respect to any claim arising out of or in connection with the use of this instrument (even if either or both of Dr. Richard Shulman and THP have been advised of the possibility of such loss or damage in advance).

The Sour Seven was developed for the purpose of open access distribution to be used freely among researchers, clinicians, allied health staff, and all caregivers.
